# Physiotherapy Management Post-operative to Total Extensor Hallucis Longus Rupture: A Case Report

**DOI:** 10.7759/cureus.50434

**Published:** 2023-12-13

**Authors:** Akanksha R Hege, Chitrakshi Choubisa, Neha Chitale, Pratik Phansopkar

**Affiliations:** 1 Musculoskeletal Physiotherapy, Ravi Nair Physiotherapy College, Datta Meghe Institute of Higher Education and Research, Wardha, IND

**Keywords:** extensor hallucis longus, post-operative physiotherapy management, physiotherapy, case report, functional rehabilitation, physiotherapy management, laceration, extensor hallucis longus tendon rupture

## Abstract

Due to the contracture or degeneration of the ruptured tendon, using primary suturing to treat chronic extensor hallucis longus (EHL) tendon ruptures is challenging. The most common cause of EHL rupture is a laceration when a sharp object drops over the dorsum of the foot. EHL injuries are rare cases. In this report, we present a case of a 23-year-old female who was operated on for EHL rupture; she visited the Physiotherapy department with complaints of pain swelling over the left dorsum of the foot and was unable to perform great toe movements for six weeks. After three weeks of patient-tailored rehabilitation protocol that included interventions like faradic stimulation, strengthening exercises, gripping exercises, proprioception training, etc., we assessed the patient’s primary outcomes like pain intensity, muscle strength, and range of motion and functional outcome measures that included lower extremity functional scale score at the end. Improvement in all the outcomes was seen. Our case report concludes that physiotherapy intervention has improved the primary and secondary outcomes and has helped patients to perform functional activities efficiently, such as maintaining balance while standing, walking, and bearing equal weight. This case report portrays that early physiotherapy treatment post-operatively in EHL rupture cases proves to be very beneficial.

## Introduction

Extensor hallucis longus (EHL) injuries are one of the least prevalent injuries. As EHL tendon ruptures are rare, the literature on the rehabilitation of such injuries is lacking [1]. EHL muscle originates from the mid-third of the anterior aspect of the fibula, medial to the origin of the extensor digitorum longus, and its tendon attaches to the distal phalanx of the great toe [2]. EHL rupture can lead to a gradual contracture deformity of the hallux [1]. The most common cause of EHL rupture is laceration when a sharp object drops over the dorsum of the foot. Conservative and surgical treatments are available for the condition. It has been found that conservative treatment comes with the complication of recurrent rupture, which can be prevented using early physiotherapy management and rehabilitation [3]. Physiotherapeutic rehabilitation plays an important role in the condition of EHL laceration and has shown improvement in the patient’s range, strength, and lower extremity function. It also concludes that the patient fully recovered [4]. Electrotherapy modalities like short-duration interrupted current, i.e., faradic current, play a significant role in the strengthening of weakened muscles, which in response helps in improving the range of motion [5]. Since the foot is a vital constituent in maintaining the equilibrium of the person’s anatomical framework, an injury to the foot or ankle might eventually result in secondary complications and, hence, should be treated immediately and effectively [6]. As the literature regarding physiotherapy rehabilitation is insufficient, this case report provides a thorough protocol for the rehabilitation and improving the patient's functional abilities. In this case report, we present a case of a 23-year-old female who underwent EHL repair and came to the Physiotherapy department for rehabilitation after four weeks.

## Case presentation

Patient information

**Figure 1:**
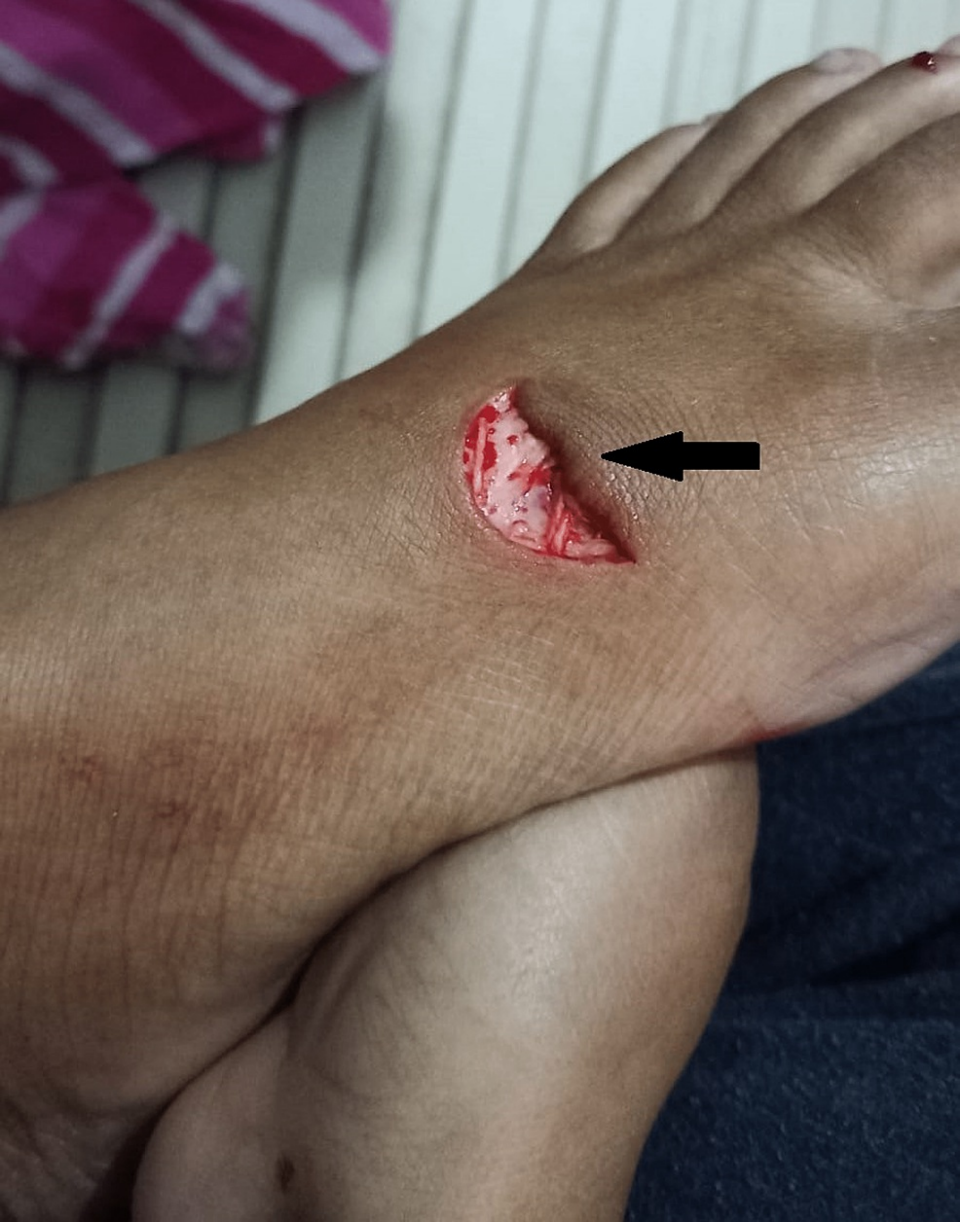
Laceration on the dorsal aspect of the foot

A 23-year-old female, presented with complaints of pain, swelling over the left dorsum of the foot, and inability to perform great toe movements for six weeks. The patient gave an alleged history of broken glass fall over a left foot as shown in Figure 1. Immediately after the trauma, the patient developed pain that was sudden in onset and constant, scoring 9/10 on Numerical Pain Rating Scale (NPRS); the pain was aggravated by movements and was relieved on rest and medication. She faced difficulty in weight-bearing and the extension of her toes on the affected side. The patient’s personal, medical, family, and psycho-social histories were not significant. The patient was operated on for EHL rupture, three days after the incident. After four weeks, the patient came to the Physiotherapy Outpatient department for rehabilitation.

Clinical presentation

The patient’s consent was taken prior to the examination. The patient was conscious, was well acquainted with her surroundings, her body build was mesomorphic, and she had stable vitals. The pain was 8/10 on NPRS; pain history consisted of sudden onset of pain, with gradual progression, dull aching, aggravation on movement of ankle and toes, and relief on rest and medications. The patient was observed in a supine position. On inspection, swelling over the left foot was present; a hypertrophic scar was present of size 2x1 cm over the dorsal surface of the great toe of the left foot. Grade II (patient complains of pain and winces) tenderness was present. On examination, flexion and extension ranges of the great toe were reduced, Manual Muscle Testing of great toe extensors was grade I, i.e., flickering of contractions was present, and tightness of the Achilles tendon and hamstring was present in the left leg.

Diagnostic assessment

**Figure 2:**
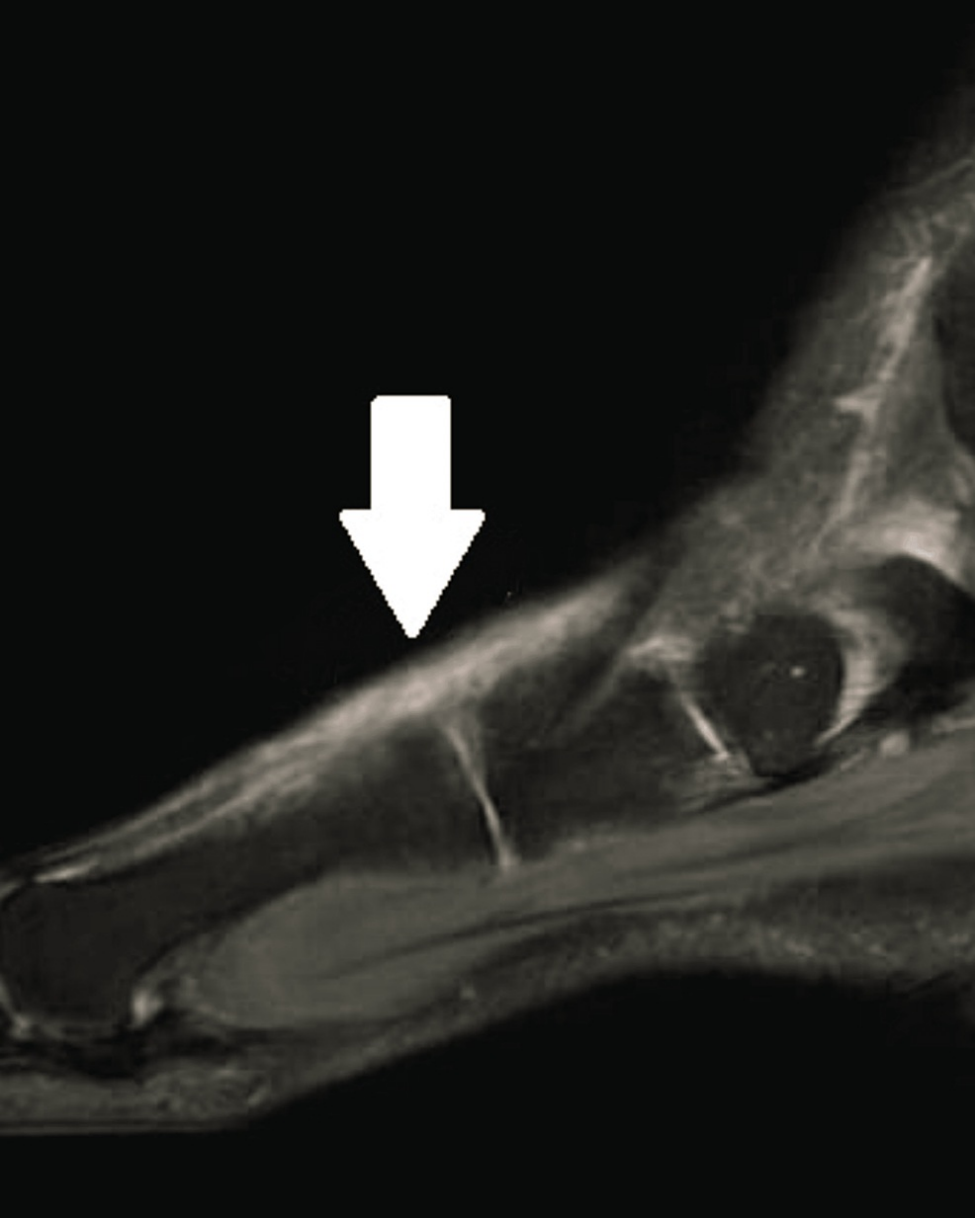
MRI showing extensor hallucis longus rupture

MRI was suggested for evaluation. MRI findings showed that there was disruption of fibers of extensor hallucis longus tendon and there was fluid collection around it. There was a disruption of fibers of the extensor hallucis longus tendon with a high-grade tear as shown in Figure 2.

Physiotherapy management

**Figure 3:**
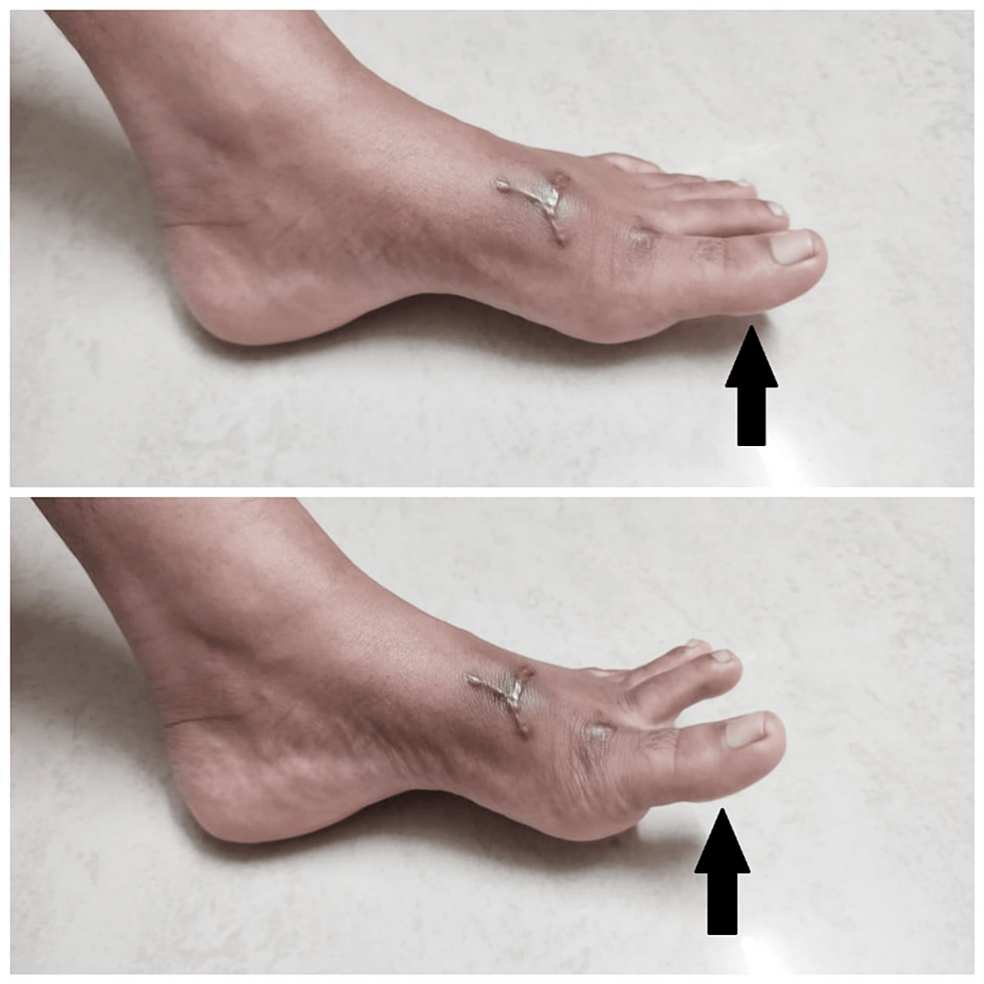
Progression of extension of toes A (top): First day of physiotherapy treatment; B (bottom): Third week of physiotherapy treatment

**Figure 4:**
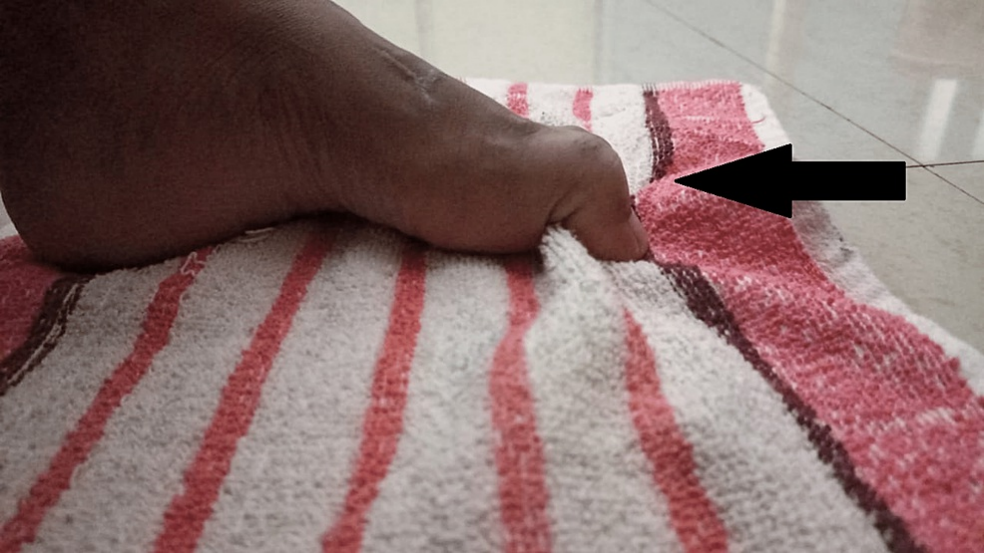
Strengthening of intrinsic muscles by griping exercises

Physiotherapy management was initiated after four weeks of surgical repair of extensor hallucis longus rupture. After proper evaluation, a planned physiotherapy protocol was designed according to the findings of the evaluation. The patient visited the Physiotherapy department five days a week for three weeks. The patient was motivated throughout the span of treatment and tolerated the treatment well. The physiotherapy management is described in Table 1 and the physiotherapy management and the progression of the patient during and after management are shown in Figure 3A, Figure 3B, and Figure 4.

**Table 1:** Physiotherapy management IFT: interferential therapy, US: ultrasound, MTP: metatarsophalangeal, IP: interphalangeal, SLR: straight leg raise, reps: repetitions, kg: kilogram *BOSU®, Ashland, Ohio, United States; **THERABAND Professional Latex Resistance Bands (Performance Health, Warrenville, Illinois, United States)

Goals	Physiotherapy intervention	Treatment regime
To reduce pain and edema	Rest, ice, compression, elevation	10 minutes for 3-4 times a day for 2 weeks.
	Combination therapy (IFT + US)	10 minutes for 1^st^ week, 7 minutes for 2^nd^ week.
To improve mobility of the ankle, MTP, and IP joints.	Stimulation of extensor hallucis longus using short-duration interrupted current (faradic current).	3 sets of 15 contractions for two weeks simultaneous active range of motion of 1^st^ MTP joint.
	Active range of motion exercises of toes and ankle.	In week 1-2, 10 reps x 2 sets and in week 2-4, 15 reps x 3 sets
To improve the strength of intrinsic, great toe extensors, proximal muscles	Implementing griping exercises for strengthening of foot intrinsic muscles (Figure 3)	In weeks 1-2, 10 reps x 10-sec hold, and in weeks 2-4, 10 reps x 15-sec hold.
	Muscle energy technique for great toe extensors	Isotonic contractions initially with 25% resistance and gradually increase resistance by 50% and later on by 75%.
	Dynamic quadriceps strengthening, SLR, Bridging	In week 1, 10 reps x 5-sec holds, in week 2, 10 reps with half kg weight cuff and bridging using red THERABAND** and in weeks 3-4, 10 reps with 1 kg weight and bridging using green THERABAND.
To improve weight-bearing, proprioception, and balance on the affected side	Weight-bearing by initiating single-leg standing, step up-step down, squatting, forward lunges	Initiating partial weight from week 2 and proceeding with full weight bearing by 3^rd^ week and 4^th^ week (10 reps x 1 set).
	Proprioception and balance training using a balance disc and Bosu ball*	Initiating from 3^rd^ week for 1 minute x 3 sets.
To prevent tissue adhesions, collagen synthesis	Scar mobilization	For 5 minutes, thrice a day.
To improve activities of daily living	Advise partial weight bearing while standing and walking	For 1-2 weeks.
	Educating stair climbing, while climbing upstairs put the non-affected extremity first, and while stepping down put the affected extremity first	For 1-2 weeks.

Follow-up and outcome measures

Initially, the patient was treated on a daily basis in the Outpatient department for three weeks and follow-up was advised after every 15 days. Outcome measures are mentioned in Table 2.

**Table 2:** Outcome measures NPRS: numerical pain rating scale, MTP: metatarsophalangeal joint, Grade I: flickering contraction, Grade III: complete range of motion against gravity, Grade IV: complete range of motion against minimal resistance

Outcome measures	Firstday of treatment		After two weeks of treatment		After three weeks of treatment	
NPRS	8/10		6/10		3/10	
Manual Muscle Testing						
1^st ^Interphalangeal dorsiflexors	Grade I		Grade III		Grade IV	
1^st ^Interphalangeal planterflexors	Grade I		Grade III		Grade IV	
1^st^ MTP dorsiflexors	Grade I		Grade III		Grade IV	
1^st^ MTP planterflexors	Grade I		Grade III		Grade IV	
Ankle dorsiflexors	Grade I		Grade III		Grade IV	
Ankle planterflexors	Grade I		Grade III		Grade IV	
Range of Motion						
Movement	Passive	Active	Passive	Active	Passive	Active
1^st ^Interphalangeal flexion	0^0^-10^0^	0^0^	0^0^-35^0^	0^0^-11^0^	0^0^-48^0^	0^0^-37^0^
1^st ^Interphalangeal extension	10^0^-0^0^	0^0^	35^0^-0^0^	11^0^-0^0^	48^0^-0^0^	37^0^-0^0^
1^st^ MTP flexion	0^0^-05^0^	0^0^	0^0^-14^0^	0^0^-12^0^	0^0^-27^0^	0^0^-24^0^
1^st ^MTP extension	05^0^-0^0^	0^0^	14^0^-0^0^	12^0^-0^0^	27^0^-0^0^	24^0^-0^0^
Lower Extremity Functional Scale Score	28/80		50/80		65/80	

## Discussion

EHL injuries are infrequent cases, and studies regarding their rehabilitation are even rarer. Joseph et al. reported a rehabilitation protocol in their case report for EHL laceration; he gave controlled active and passive mobilization therapy, gait training, and bracing and noticed an improved range of great toe extension, EHL muscle strength, and overall lower extremity function [7]. The improvement was marked in the range of motion from 50^o^ to 73^o^ of active dorsiflexion, from 70^o^ to 85^o^ of passive dorsiflexion, the strength of hallux metatarsophalangeal (MTP) and interphalangeal (IP) joint muscles from 4/5 and 3/5 to 5/5 to 4+/5, a manifestation of improvement was observed in Shortform-36 (SF-36) scoring along with full functional recovery [8,9]. Goldsmid in his study proved that faradic current stimulation improved the strength of the extensors of the hand [10]. A study states that to regain joint function and muscle strength, patients should enroll in physiotherapy management by observing their excellent outlook of the patient. Bronner et al. stated in their study that physiotherapy management has shown significant improvement in range, the strength of MTP and IP joint and muscles of the great toe, and functional activity in post-operative extensor hallucis longus tendon repair [11]. Our study has also given similar significant results. In this study, pain management has reduced the intensity of pain from 8 to 3; muscle strength has been improved from grade I to grade IV, and the range of motion has also been increased by around 30-35%. The intervention was well planned according to the patient's complaints and the positive findings in the assessment. There was a significant improvement in the patient's functional activities.

## Conclusions

In the case of post-operative extensor hallucis longus tendon repair, a physiotherapy rehabilitation program plays a significant role. Since the foot is the foundation of almost all dynamic activities of daily living, it is important to maintain its integrity. This study shows that physiotherapy intervention has improved the primary and secondary outcomes and has helped the patient to efficiently perform functional activities such as maintaining balance while standing, walking, and equal weight bearing.
